# Isolation, Identification, and Characterization of *Colletotrichum falcatum* and *Fusarium madaense* Associated with Sugarcane Red Rot Disease in Southwest China

**DOI:** 10.3390/microorganisms14061280

**Published:** 2026-06-05

**Authors:** Jie Li, Xiaoyan Wang, Rongyue Zhang, Yinhu Li, Jiong Yin, Changmi Wang, Hongli Shan

**Affiliations:** Yunnan Key Laboratory of Sugarcane Genetic Improvement, Sugarcane Research Institute, Yunnan Academy of Agricultural Sciences, Kaiyuan 661699, China; lijie0988@163.com (J.L.); xiaoyanwang402@sina.com (X.W.); rongyuezhang@hotmail.com (R.Z.); liyinhu93@163.com (Y.L.); yinjiong@126.com (J.Y.); wcmlucky@163.com (C.W.)

**Keywords:** sugarcane red rot, *Colletotrichum falcatum*, *Fusarium madaense*, pathogen identification, multigene phylogeny, new report

## Abstract

Sugarcane red rot is a critical constraint threatening the stability and sustainability of sugarcane production in Southwest China, where Yunnan and Guangxi Provinces are the core cultivation regions. To provide a scientific basis for targeted disease management and ensure sugarcane production security, 40 symptomatic red rot samples were collected from 10 sugarcane varieties across 7 locations in these two provinces. A total of 57 fungal isolates were obtained, and they were identified through morphological characterization, multigene phylogenetic analysis (ITS/*ACT*/*TUB2* for *Colletotrichum* sp. and *EF-1α*/*RPB2* for *Fusarium* sp.), and pathogenicity tests on the susceptible cultivar Yuetang 93-159 using three representative isolates per species. The results show that 36 isolates were identified as *Colletotrichum falcatum* and divided into light and dark morphotypes. Phylogenetic analysis revealed that Yunnan and Guangxi isolates clustered in Clade I and Clade II, respectively. The remaining 21 isolates were identified as *Fusarium madaense*, and no sequence polymorphisms were detected in either *EF1α* or *RPB2* among these isolates, which clustered with the *F. madaense* strain isolated from sugarcane in Brazil. Pathogenicity tests on leaf midribs and stalks of this cultivar showed that the representative isolates of *C. falcatum* and *F. madaense* induced typical red rot symptoms consistent with field observations. Among the representative isolates tested, preliminary findings suggest that light-type *C. falcatum* isolates were more virulent than dark-type ones, and the *C. falcatum* isolates Cf16 and Cf1 showed higher stalk virulence than the tested *F. madaense* isolates. To our knowledge, this is the first report of *F. madaense* causing typical red rot symptoms on sugarcane in China.

## 1. Introduction

Sugarcane (*Saccharum* spp. hybrids) is an important sugar and energy crop widely cultivated in tropical and subtropical regions worldwide, particularly in Brazil, India and China [1]. It is one of the main sources of sugar, accounting for more than 85% of the total sugar production in China, and Guangxi and Yunnan Provinces are the main sugar production regions [2,3]. Due to the hot climate and perennial cultivation of sugarcane being conducive to disease development, it is susceptible to several fungal diseases affecting roots, stalks and leaves in the field, of which red rot caused by *Colletotrichum falcatum* is one of the devastating diseases of and a major constraint on sugarcane production [4,5,6]. Red rot is one of the oldest diseases of sugarcane. It was first reported in Java by Went (1893) [7] and has been found in 68 sugarcane-growing countries worldwide [8]. It has been reported as a damaging disease of sugarcane in India, Pakistan, Bangladesh, Brazil, Thailand, the United States, and China [4,5,6,8,9,10,11,12,13,14]. The loss in cane yield can be 29% with sugar recovery loss up to 31%, and under conditions favorable for the development of red rot, almost 100% loss of cane yield in seriously affected fields were reported [4,15]. In addition, sucrose inversion occurs when the stalks are infected by the fungus and reduces the total amount of recoverable sugar of the plant [11].

Red rot is reported in all sugarcane-growing areas in China, although it occurred sporadically before the 1980s and caused no threat to sugarcane production. Damaging epidemics occurred in 1993, when the disease incidence was 61.2% in severely affected fields, cane yield decreased by 15.7–30.5%, and sucrose content decreased by 27.6% in the sugarcane-growing area of Hanchuan City, Hubei Province [16]. In 2005 and 2006, red rot outbreaks caused significant yield loss in the sugarcane-growing area of Hainan Province [17]. In 2009, following a prolonged rainy season, red rot epidemics occurred on ROC22, causing significant yield losses in Zhanjiang City, Guangdong Province [18]. In 2018, the major cultivated variety ROC 1 was susceptible to red rot in Shiping County, Yunnan Province, with yield losses reaching up to 100% in severely affected fields, severely threatening sugar production output [4]. In the same year, the major cultivars Yuetang 93-159 and ROC 22 were severely infected with red rot in both the Lincang and Menglian sugarcane-planting areas of Yunnan Province, and the disease incidence was as high as 100% in severely affected fields, resulting in significant yield losses [4]. At present, because susceptible varieties have been continually planted on a large scale, coupled with outbreaks of borers, red rot has been reported in all sugarcane-planting areas and caused significant yield losses in China [4,19].

Most recently, preliminary studies have shown that other fungal species were isolated from red rot-infected sugarcane. In addition to *C. falcatum*, *Fusarium* species such as *F. sacchari*, *F. proliferatum* and *F. madaense* were reported to induce symptoms of red rot in sugarcane [20,21]. Accurate identification of the causal organism is vital in disease management and plant breeding programs. The objective of this study was to preliminarily identify the pathogens causing red rot from different sugarcane varieties in the main sugarcane production areas of Guangxi and Yunnan Provinces, China. We combined morphological characterization, molecular phylogenetic analysis, and pathogenicity tests to clarify the disease etiology. The results from this study could establish a scientific foundation for the management and development of disease-resistant varieties of red rot in China.

## 2. Materials and Methods

### 2.1. Sample Collection, Fungal Isolation and Pure Culture Maintenance

Sugarcane stalks and leaf midrib samples were collected in 2022 from plants in the field from 10 varieties exhibiting symptoms of red rot. From each field, four symptomatic stalks or leaves were collected from diseased plants of the same variety within the field as one sample. Samples were obtained from seven cities/counties in two major sugarcane-producing provinces in China, including (1) Lincang City, (2) Puer City, (3) Kaiyuan City, (4) Mile City and (5) Menghai County of Yunnan Province and (6) Hechi and (7) Wuzhou City of Guangxi Province (Table 1). The red rot-symptomatic samples of sugarcane cultivars included six varieties (Yuetang 93-159, Yunzhe 081609, Funong 10-14405, ROC 22, Chuantang 79-15 and Yingyu 91-59) from Yunnan and four varieties (Guitang 44, Guitang 42, Liucheng 05-136, and Liucheng 03-1137) from Guangxi in this study. Symptomatic tissues were taken from the margins of lesions; cut into 5 × 5 mm pieces; surface-sterilized with 75% ethanol (Tianjin Fuyu Fine Chemical Co., Ltd., Tianjin, China) for 30 s, followed by 2% NaOCl (Nanjing Chemical Reagent Co., Ltd., Nanjing, China) for 2 min; rinsed three times with sterile water; air-dried on sterile filter paper; and then transferred to potato dextrose agar (PDA, Guangdong Huankai Microbial Sci. & Tech. Co., Ltd., Guangdong, China) medium and incubated at 25 °C in the dark. Pure isolates were obtained by transferring hyphal tips onto fresh PDA plates and, using the single-spore isolation technique, and maintained on PDA plates with periodic sub-culture and stored in a refrigerator at 4 °C [5].

### 2.2. Morphological Characterization

Thirty-six *Colletotrichum* isolates were cultured on PDA in the dark at 25 °C for 14  days for cultural characterization (colony color and mycelium). For morphological observation, conidia, appressoria, setae, and acervulus were recorded, and the shape and size of the conidia (50 per isolate) from three representative isolates were measured [22]. Twenty-one *Fusarium* isolates were incubated on synthetic nutrient-poor agar (SNA, Coolaber Science & Technology, Beijing, China) and PDA plates for 7 days at 25 °C in the dark for cultural characterization (colony color, mycelium and pigmentation) [23,24]. Micromorphological characteristics (conidiophores, microconidia and macroconidia, and chlamydospores) were observed after 14 days of incubation under a 12/12 h near-ultraviolet light/dark cycle at 25 °C on SNA, and their shape and size (50 per isolate) from three representative isolates were measured using a light microscope (Leica DM4, Leica Microsystems, Wetzlar, Germany) [25].

### 2.3. Molecular Identification of the Pathogen

For genomic DNA extraction, 57 isolates were grown on PDA in the dark at 28 °C for 7 days. Fresh mycelia were scraped from the surface of each colony. The genomic DNA was extracted using the Ezup Column Fungi Genomic DNA Purification Kit (Sangon Biotech, Shanghai, China) according to the manufacturer’s instructions. DNA products were stored at −20 °C. For *Colletotrichum* isolates, DNA sequences were amplified by PCR using the internal transcribed spacer region (ITS), Actin (*ACT*), and *β-tubulin* (*TUB2*) with the primer pairs ITS1/ITS4 [26], ACT-512F/ACT-783R [27], and Bt-1/Bt-2 [27,28,29], respectively. For *Fusarium* isolates, fragments of the translation elongation factor-1α gene (*EF-1α*) and the second largest subunit of RNA polymerase II (*RPB2*) were amplified by PCR with the primer pairs EF1/EF2 [30], 5F2/7cR and 7cf/11ar, respectively [31,32]. The primers used in this study were synthesized by Sangon Biotech (Shanghai, China).

The PCR was performed with a volume of 25 µL containing 2.5 µL of DNA template, 1 µL (10 µM) of each primer, 12.5 µL of 2 × EasyTaq PCR SuperMix (TransGen Biotech, Beijing, China) and 8 µL of nuclease-free water. The amplification conditions were as follows: initial denaturation at 94 °C for 4 min, followed by 35 cycles of denaturation at 94 °C for 30 s; annealing at 52 °C (ITS), 58 °C (*ACT*), 55 °C (*TUB2* and *EF-1α*), or 60 °C (*RPB2*) for 30 s; extension at 72 °C for 45 s; and a final extension at 72 °C for 7 min. The amplified products were analyzed by electrophoresis on a 1.5% agarose gel and stained with GoldView (Solarbio, Beijing, China). DNA sequencing was done by the BGI Sequencing Co., Ltd. (Beijing, China).

### 2.4. Phylogenetic Analysis

The obtained sequences were deposited in GenBank, and the accession numbers are listed in Table 1. Sequencing results obtained in this study were subjected to BLAST analysis in GenBank (https://blast.ncbi.nlm.nih.gov), and reference species sequences used for phylogenetic analyses were also obtained from GenBank and are listed in Table 2. The sequences were aligned using the DNAMAN 8 software (Lynnon Biosoft, San Ramon, CA, USA) and concatenated in the Sequence Matrix v1.9 program. The evolutionary history was inferred using the neighbor-joining (NJ) and maximum likelihood (ML) methods for concatenated sequences. NJ and ML analyses were performed in the Molecular Evolutionary Genetics Analysis (MEGA) software version 6 under the Kimura 2-parameter model [33,34] with 1000 bootstrap replications [34,35] and manually adjusted to allow for maximum sequence similarity [35]. In this study, phylogenetic analyses performed by both NJ and ML methods produced similar topologies, so only the phylogenetic tree constructed by the ML method is presented.

### 2.5. Pathogenicity Tests

Pathogenicity tests were performed on healthy six-month-old potted plants of Yuetang 93-159, a susceptible and major cultivated sugarcane variety. Representative isolates of *C. falcatum* (Cf1, Cf16 and Cf33) and *F. madaense* (FM2, FM9 and FM6) were selected for pathogenicity tests and grown on PDA at 25 °C for 14 days. Two methods of inoculation were used. In the first, sugarcane leaf midribs of each plant were wounded with a sterile needle, inoculated using 8 mm mycelial agar plugs from each representative isolate, and covered with wet cotton to maintain high relative humidity [20]. Sterile PDA plugs were used as controls. Disease symptoms were observed and recorded at 7 days post-inoculation, and lesion lengths were measured with a ruler to assess disease severity and evaluate virulence. In the second method, the inoculum of each representative isolate was adjusted to a concentration of 1 × 10^6^ conidia/mL in sterile water by a hemocytometer. A hole was made using a cork borer on the 3rd internode from the base of the stalk, and 100 µL of spore suspension was injected into the hole of each sugarcane plant using a pipette [5]. The punctured point was sealed with plastic wrap to prevent entry of insects and secondary infections. Plants inoculated with sterile distilled water via the same method were used as controls. Stalks were cut longitudinally to observe internal symptoms 30 days after inoculation. Disease severity was assessed by visual observation of internal lesion transgression of the inoculated node. Plants were placed in a greenhouse at 25 ±  2 °C. For each isolate, three plants were inoculated per replicate, and the experiment was independently repeated three times, for a total of nine plants tested per isolate (3 plants × 3 replicates). Every fungal isolate included in the pathogenicity tests was re-isolated from inoculated leaves and stalks using the same method used above to confirm its identity by morphological and molecular techniques as described above.

### 2.6. Statistical Analyses

Statistical analyses were performed using the GraphPad Prism 8.0 software (GraphPad Software, San Diego, CA, USA). All data were first tested for normality (Shapiro–Wilk test) and homogeneity of variances (Levene’s test). As not all datasets met the assumptions of parametric ANOVA, the non-parametric Kruskal–Wallis test was subsequently applied. Conidia size and lesion length data from the pathogenicity test were analyzed using the Kruskal–Wallis test, followed by Dunn’s post hoc test. For the stalk assay, the indicator for lesion transgression across nodes is an ordinal scale of 0–4. For conidial size, comparisons were performed only within *C. falcatum* isolates or within *F. madaense* isolates; no cross-genus comparisons were made. The significance level was set at *p* < 0.05. All data are expressed as the mean ± standard deviation (SD).

## 3. Results

### 3.1. Fungal Isolation

**Figure 1:**
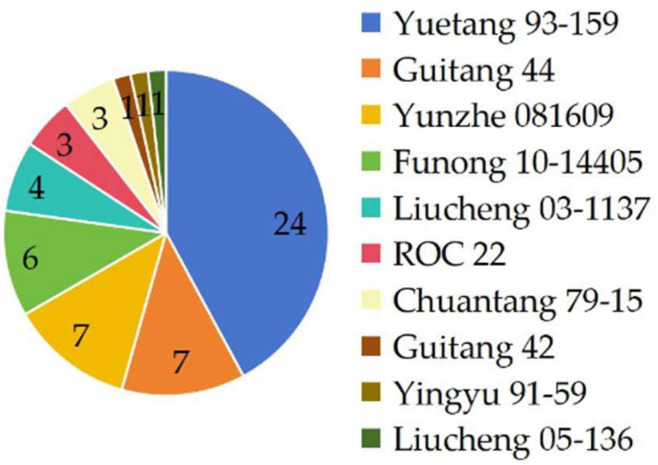
Number of isolates obtained from ten sugarcane varieties.

A total of 57 isolates were obtained in this study, of which 44 were from Yunnan Province and 13 from Guangxi Province. Among the ten sugarcane varieties, isolates were most frequently obtained from Yuetang 93-159 (24 isolates); followed by seven isolates each from Guitang 44 and Yunzhe 081609; six from Funong 10-14405, four from Liucheng 03-1137; three each from ROC 22 and Chuantang 79-15; and one each from Yingyu 91-59, Guitang 42, and Liucheng 05-136 (Figure 1).

### 3.2. Morphology Characteristics of Isolates

**Figure 2:**
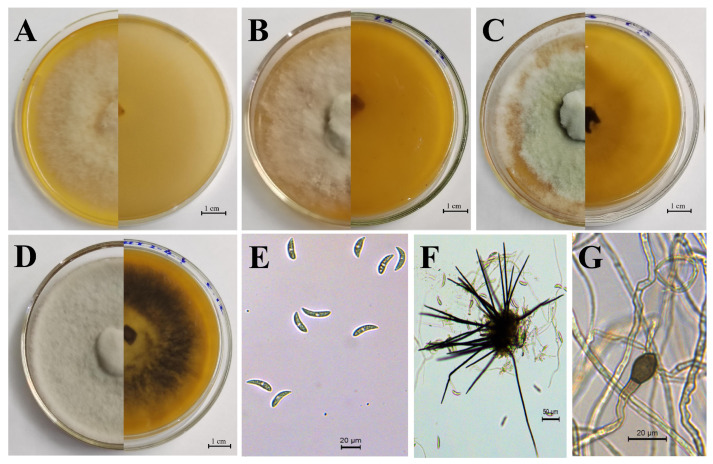
Morphological characteristics of *C. falcatum* isolated from red rot of sugarcane. (**A**–**D**) Top and underside view of *C. falcatum* on PDA. (**E**) Conidia. (**F**) Setae. (**G**) Appressoria. Scale bars: (**A**–**D**) = 1 cm, (**E**,**G**) =20 μm, and (**F**) = 50 μm.

The morphological characterization revealed the presence of two fungal species. A total of 36 isolates were identified as *Colletotrichum* sp. and 21 isolates were putatively known as *Fusarium* sp. In this survey, *Colletotrichum* sp. was the most frequently isolated genus, accounting for 63.2% (36 of 57) of the total isolates. According to colonies and morphological features on PDA, the 36 *Colletotrichum* sp. isolates were classified into light and dark types. The isolates in group 1 (the light type) were greyish white or whitish grey, with sparse aerial mycelium, colonies were mostly raised and fluffy, conidial masses were salmon pink, and there was medium to high sporulation (Figure 2A,B). The isolates in group 2 (the dark type) were greyish green or dark grey, with abundant aerial mycelium, colonies were mostly flat and less fluffy, and there was poor sporulation (Figure 2C,D). Group 1 comprised 25 isolates, and group 2 comprised 11 isolates (Table 1). Conidia were falcate or sickle-shaped, contents were granular and sometimes contained oil globules, and the size of all the isolates ranged between 26.4 and 38.3 × 5.3–6.9 µm (Figure 2E). Setae were black and sparse (Figure 2F). Appressoria were globose or clavate, and medium brown (Figure 2G). These morphological characteristics were similar to *C. falcatum* [21], while species identification was confirmed by multigene phylogenetic analysis. The morphological and colony features of three representative isolates (Cf1, Cf16 and Cf33) are described in Table 3. The light type (Cf1 and Cf16) produced more conidia than the dark type (Cf33), and significant differences in conidial size among isolates were determined by the Kruskal–Wallis test with Dunn’s post hoc test (*p* < 0.05), as denoted by different superscript letters in the table.

**Figure 3:**
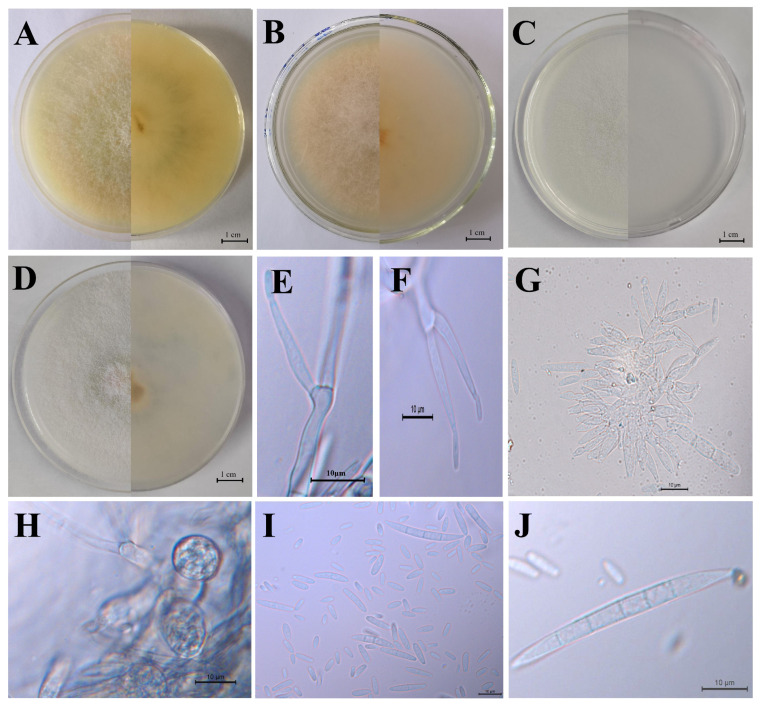
Morphological characteristics of *F. madaense* isolated from red rot of sugarcane. (**A**–**D**) Top and underside view of *F. madaense* on SNA. (**E**,**F**) Conidiophores and phialides. (**G**) Sporodochium phialides. (**H**) Chlamydospores. (**I**) Microconidia and macroconidia. (**J**) Macroconidia. Scale bars: (**A**–**D**) = 1 cm and (**E**–**J**) = 10 μm.

Colonies of *Fusarium* sp. on PDA were white to pale salmon, flat, floccose aerial mycelium, and pigmentation of the reverse side of the colony was also white to lavender gray to pale salmon (Figure 3A,B). Colonies of *Fusarium* sp. on SNA were white and velvety, with scarce aerial mycelium without pigmentation (Figure 3C,D). Conidiophores on aerial mycelium were straight or flexuous, septate, hyaline, smooth and thin-walled; simple or reduced to conidiogenous cells; and bore terminal single monophialides. Phialides were subulate to subcylindrical, smooth and thin-walled (17.8–38.6 × 2.5–3.5 µm) (Figure 3E,F). Sporodochium phialides were doliiform to subcylindrical smooth and thin-walled (9.2–15.4 × 3.0–4.0 µm) (Figure 3G). Chlamydospores present on SNA were globose to subglobose, hyaline, solitary, smooth and thick-walled (7.5–13.3 × 5.8–11.7 µm) (Figure 3H). Microconidia were produced in long chains at the tip of monophialides and were hyaline, ellipsoidal or clavate, smooth and thin-walled, with 0–3-septate and mostly 0 septate (4.2–22.9 × 1.7–4.2 µm) (Figure 3I). Macroconidia were lunate to falcate, tapered towards apical and basal ends, hyaline, smooth and thin-walled, with 3–5-septate and mostly 3 septate (25.8–52.5 × 3.3–4.6 μm) (Figure 3I,J). The cultural and morphological characteristics of the colony and conidia matched the description of *F*. *madaense* [36,37]. The morphological and colony features of three representative isolates (FM2, FM6 and FM9) are described in Table 3, and there are no significant differences among the isolates.

### 3.3. Molecular Identification

**Figure 4:**
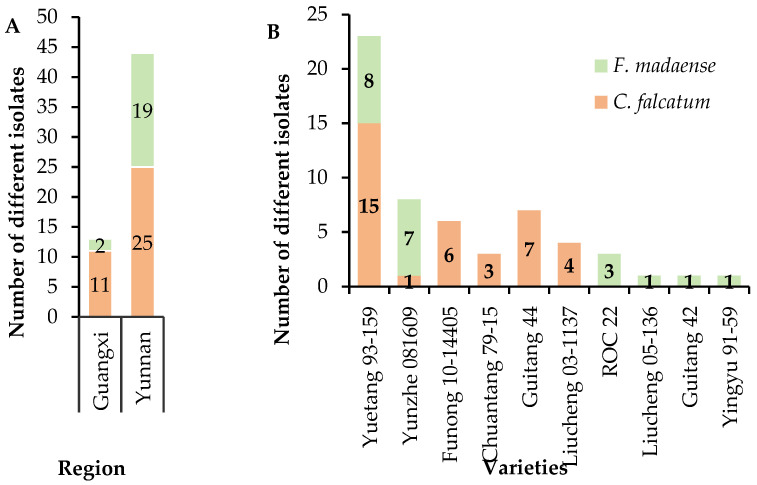
Proportions of *C. falcatum* and *F. madaense* isolated from sugarcane in China. (**A**) Number of *C. falcatum* and *F. madaense* isolates collected from Yunnan and Guangxi. (**B**) Number of *C. falcatum* and *F. madaense* isolates collected from ten sugarcane varieties.

The aligned sequences of ITS (565 bp), *ACT* (277 bp) and *TUB2* (770 bp) were obtained from 36 *Colletotrichum* sp. isolates for further molecular identification. GenBank BLAST searches with DNA sequences of ITS (OR523414–OR523449) confirmed that the nucleotide sequences of these isolates showed 99–100% nucleotide identity with the *C. falcatum* isolates COUFAL0263–COUFAL0266 (MT796068–MT796071) from Brazil and the isolate SUCF04 (MT197390) from Pakistan. BLAST searches of the *ACT* (OR542860–OR542895) sequences also revealed 99–100% identity with the *ACT* sequences of the *C. falcatum* isolates CML 4080 (MW455492) from Brazil. BLAST searches of the TUB2 (OR542896–OR542931) sequences shared 99–100% identity with the *TUB2* sequences of the *C. falcatum* isolates COUFAL0263–COUFAL0266 (MT778880–MT778883) from Brazil. The 36 isolates were identified and confirmed as *C. falcatum* using sequence data of ITS, *ACT* and *TUB2* genes, including 25 isolates from Yunnan Province and 11 from Guangxi Province (Figure 4A). Among these isolates, *C. falcatum* was most frequently isolated from the cultivar Yuetang 93-159 (15 isolates), followed by Guitang 44 (seven isolates), Funong 10-14405 (six isolates), Liucheng 03-1137 (four isolates), Chuantang 79-15 (three isolates), and Yunzhe 081609 (one isolate) (Figure 4B).

The sequences of the *EF-1α* (657 bp) and *RPB2* (1806 bp) genes were obtained from 21 *Fusarium* sp. isolates for further molecular identification. The *EF-1α* (PP796366–PP796386) and *RPB2* (PP691538–PP691558) nucleotide sequences showed 99.62% and 99.88% sequence similarity to the type strain CBS 146669 (*EF-1α*: MW402098, *RPB2*: MW402764) of *F. madaense*, respectively, and 99.88% and 100% sequence similarity to the strain CML 3586 of *F. madaense* (*EF-1α*: MH187929, *RPB2*: MH187912), respectively. No sequence polymorphisms were observed within *EF-1α* and *RPB2* DNA loci among *F. madaense* isolates obtained in this study, and all isolates yielded identical sequences at all loci. The 21 isolates were identified as *F. madaense* based on BLAST searches of the two loci, and 19 isolates were from Yunnan Province and two from Guangxi Province (Figure 4A). Among these isolates, *F. madaense* was also most frequently isolated from the cultivar Yuetang 93-159 (eight isolates), followed by Yunzhe 081609 (seven isolates), ROC 22 (three isolates), Guitang 42 (one isolate), Liucheng 05-136 (one isolate), and Yingyu 91-59 (one isolate) (Figure 4B).

### 3.4. Phylogenetic Analysis

**Figure 5:**
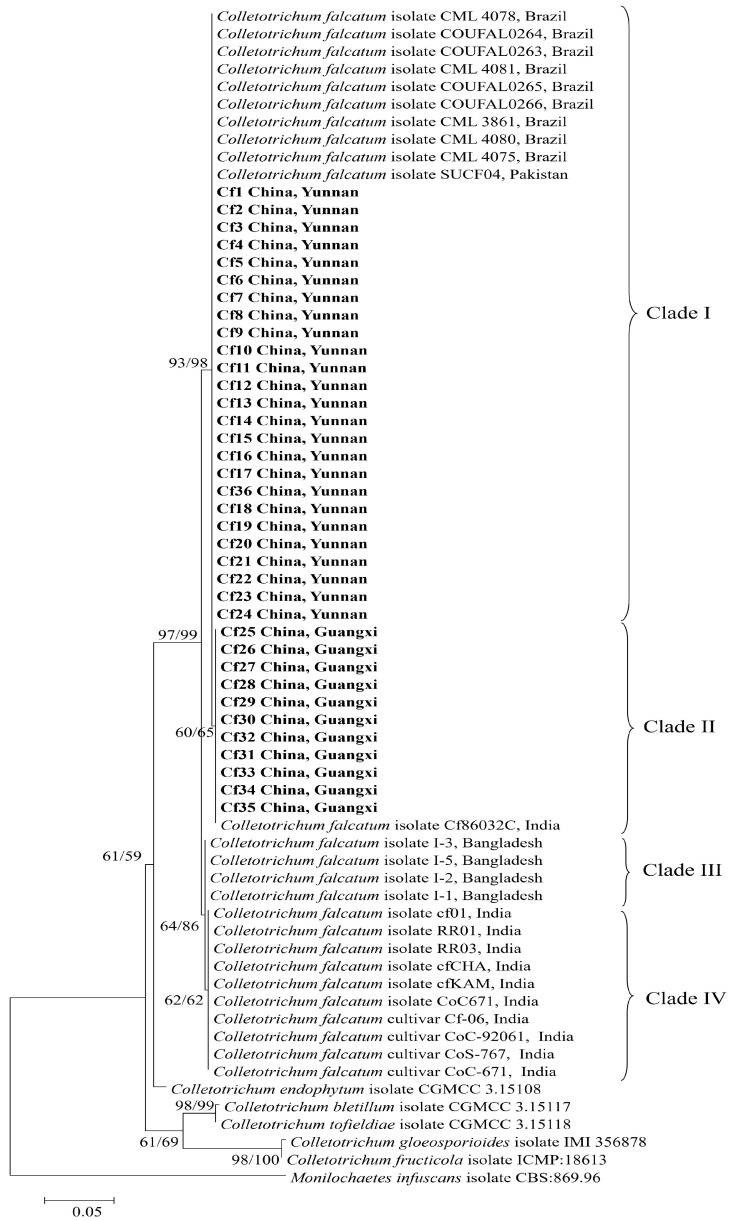
Phylogenetic tree of *C. falcatum* based on concatenated ITS, *ACT* and *TUB2* sequences, constructed using neighbor-joining and maximum likelihood. Bootstrap support values (1000 replicates) ≥ 50% are shown at the nodes as NJ/ML. Isolates obtained in this study are indicated in bold.

The phylogram constructed from the combined ITS, *ACT* and *TUB2* genes revealed that the 36 *Colletotrichum* sp. isolates obtained in this study and 25 *C. falcatum* isolates from GenBank were clustered into four clades: Clade I (Yunnan, Brazil and Pakistan), Clade II (Guangxi and one isolate from India), Clade III (Bangladesh) and Clade IV (India) (Figure 5). In Clade I, twenty-five isolates from Yunnan, China, clustered with *C. falcatum* isolates from Brazil and Pakistan, with bootstrap values of 98% NJ and 93% ML. In Clade II, 11 isolates from Guangxi, China, showed a tendency to group the *C. falcatum* isolate Cf86032C from India, with bootstrap values of 65% NJ and 60% ML. The Yunnan clade and Guangxi clade appeared as sister clades with weak to moderate support. Clade III consisted of the isolates from Bangladesh, while Clade IV consisted of the isolates from India (Figure 5). Clade III and Clade IV were rebuilt as sister clades. Thus, the phylogenetic analysis based on the combined ITS, *ACT* and *TUB2* sequences supports the identification of the 36 fungal isolates as *C. falcatum*.

**Figure 6:**
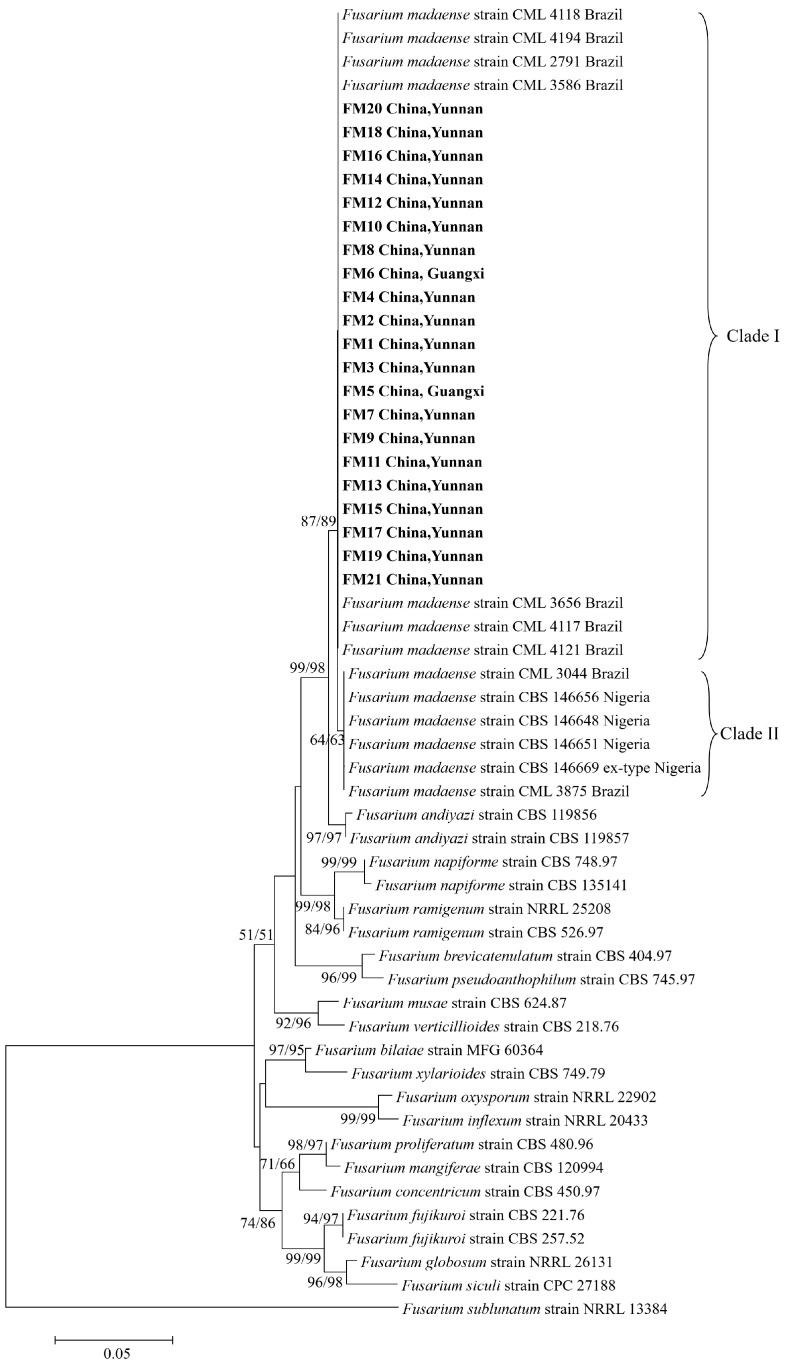
Phylogenetic tree of *F. madaense* based on the concatenated sequences of *EF-1α* and *RPB2* genes by neighbor-joining and maximum likelihood. Bootstrap support values (1000 replicates) ≥ 50% are shown at the nodes as NJ/ML. Isolates obtained in this study are indicated in bold.

The phylogram constructed from the combined *EF-1α* and *RPB2* gene sequences revealed that the 21 *Fusarium* sp. isolates obtained in this study and 13 *F. madaense* isolates from GenBank were clustered into two clades: Clade I (China and Brazil) and Clade II (Nigeria and Brazil) (Figure 6). All the 21 *Fusarium* sp. isolates from this study clustered in the same clade (Clade I) with the *F. madaense* strain (CML 3586) isolated from sugarcane in Brazil, with bootstrap values of 87% NJ and 89% ML, supporting that these 21 isolates belonged to *F. madaense* (Figure 6). Additionally, there were no sequence polymorphisms observed within these two DNA loci among the *F. madaense* isolates obtained in this study.

### 3.5. Pathogenicity Test

**Figure 7:**
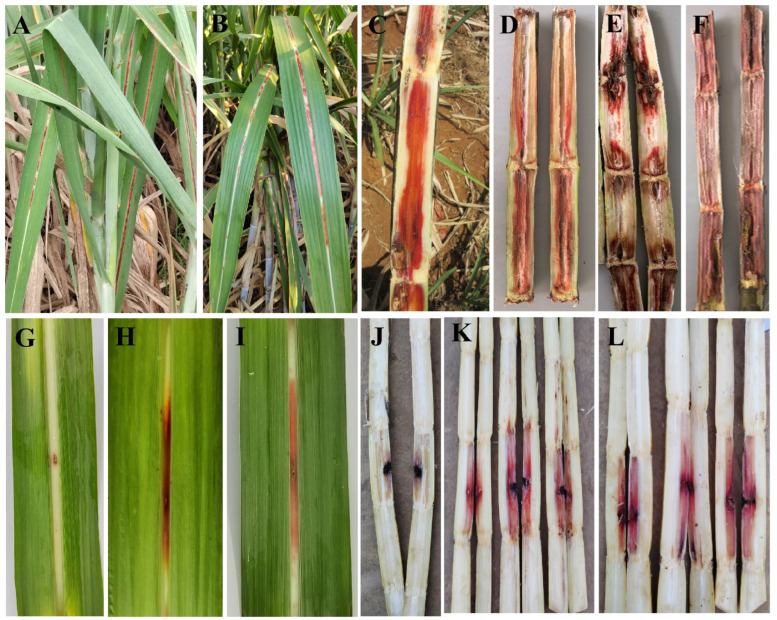
Disease symptoms of leaf midrib and stalk red rot on sugarcane plants under natural conditions and artificial inoculation. Natural infections: (**A**) Leaf midrib red rot caused by *C. falcatum*. (**B**) Leaf midrib red rot caused by *F. madaense*. (**C**) Stalk red rot caused by *C. falcatum*. (**D**–**F**) Stalk red rot caused by *F. madaense*. Artificial inoculations: (**G**) Negative control for leaf midrib. (**H**) Leaf midrib red rot inoculation with the *C. falcatum* isolate Cf33. (**I**) Leaf midrib red rot inoculated with the *F. madaense* isolate FM2. (**J**) Negative control for stalk. (**K**) Stalk red rot inoculation with the *C. falcatum* isolate Cf33. (**L**) Stalk red rot inoculated with the *F. madaense* isolate FM2.

**Figure 8:**
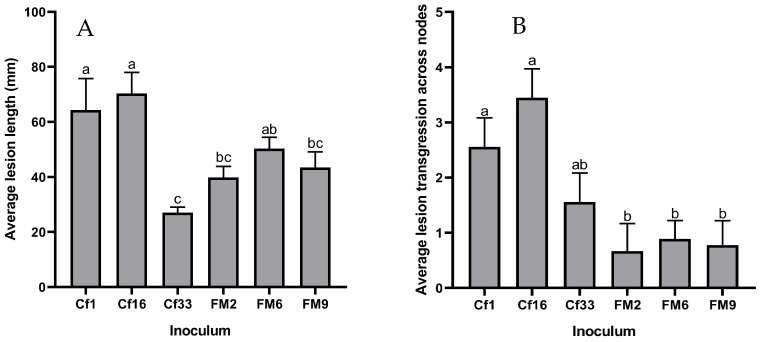
Disease development after inoculation with different strains. (**A**) Average lesion lengths (in millimeters) on sugarcane leaf midrib 7 days after inoculation with mycelial plugs of six fungal isolates. (**B**) Average lesion transgression across nodes on sugarcane stalks 30 days after inoculation with spore suspension of six fungal isolates. Columns with different letters indicate significant differences (Kruskal–Wallis test followed by Dunn’s post hoc test; *p* < 0.05).

After 2 days of inoculation, red dots appeared on the leaf midribs inoculated with the tested *C. falcatum* and *F. madaense* isolates. As the disease developed, these red dots expanded and elongated, with typical long red lesions observed 7 days post-inoculation. The symptoms exhibited in the greenhouse were similar to the red rot symptoms observed in the field, whereas the controls inoculated with sterile PDA plugs exhibited redness at the inoculation sites but no further spread of symptoms (Figure 7A,B,G–I). The Kruskal–Wallis test revealed significant differences (*p* < 0.05) in the average lesion lengths among six representative isolates, indicating variations in their virulence (Figure 8A). Post hoc comparisons using Dunn’s post hoc test showed that the isolates Cf16 and Cf1 were the most virulent, with no significant difference between them, while the isolate Cf33 was significantly less virulent than Cf1, Cf16, and FM6 (*p* < 0.05) but did not differ significantly from FM2 or FM9 (*p* > 0.05). The isolate FM6 exhibited intermediate virulence, and no significant differences were observed from Cf1, Cf16, FM2, or FM9 (*p* > 0.05), but it was significantly more virulent than Cf33 (*p* < 0.05). The isolates Cf33, FM2 and FM9 displayed lower virulence levels, with no significant difference between them (*p* > 0.05). In this experiment, the *C. falcatum* isolate Cf16 was the most virulent, with an average lesion length of 70.3 mm at 7 days post-inoculation, followed by the *C. falcatum* isolate Cf1 (mean length: 64.3 mm), the *F. madaense* isolate FM6 (mean length: 50.3 mm), the *F. madaense* isolate FM9 (mean length: 43.4 mm), the *F. madaense* isolate FM2 (mean length: 39.9 mm), and the *C. falcatum* isolate Cf33 (mean length: 27.1 mm) (Table 3).

Thirty days after inoculation with the spore suspension, the tested *C. falcatum* and *F. madaense* isolates induced typical stalk red rot symptoms in the internal tissues of the inoculated stalks, which were similar to those observed under field conditions. Only reddening occurred at the inoculation sites on stalks inoculated with sterile distilled water, and no lesion development was observed (Figure 7C–F,J–L). The Kruskal–Wallis test revealed significant differences (*p* < 0.05) in lesion transgression across nodes among the six isolates (Figure 8B). Dunn’s post hoc test showed that the isolates Cf16 and Cf1 showed the highest stalk virulence, while Cf33 showed an intermediate response with no significant difference between them (*p* > 0.05), and Cf16 and Cf1 were significantly more virulent than FM2, FM6, and FM9 (*p* < 0.05). The isolates FM2, FM6, and FM9 exhibited the lowest virulence levels, with no significant differences among them (*p* > 0.05). Among the tested isolates, the *C. falcatum* isolate Cf16 was also the most virulent (as in leaf midrib inoculation), with a lesion transgression of 3–4 nodes above the inoculated node, followed by the *C. falcatum* isolates Cf1 and Cf33, as well as the *F. madaense* isolates FM6, FM9 and FM2 (Table 3).

Pathogenicity tests showed that both *C. falcatum* and *F. madaense* induced typical red rot symptoms. The results also indicate that the light-type *C. falcatum* isolates (Cf1 and Cf16) were more virulent than the dark-type isolate (Cf33), and the *C. falcatum* isolates Cf16 and Cf1 were more virulent than *F. madaense* with inoculation of sugarcane stalks (Figure 8, Table 3). The tested fungi were re-isolated from the inoculated plants and confirmed to be the original isolates based on the morphological characteristics and molecular identification methods (*EF1α* and *RPB2* sequencing), which yielded sequences identical to those of the inoculated isolates described above; however, no fungi were isolated from the control plants. Representative isolates of *C. falcatum* and *F. madaense* fulfilled Koch’s postulates, confirming their ability to cause red rot symptom.

## 4. Discussion

Red rot commonly occurs on the leaf midribs and stalks of sugarcane, and the most damaging phase of this disease occurs when the pathogen attacks the stalk [19]. The emergence of new races has led to the breakdown of resistance in sugarcane varieties after several years of cultivation, and the disease cannot be effectively controlled by a single measure [15,38]. Many commercially popular varieties have become susceptible to red rot, such as Isd 17, Isd 18, Isd 28 and Isd 32 in Bangladesh [39]; Co 419, CoC 671, and CoJ 64 in India [15,38]; and Yuetang 93-159 and Guitang 44 in China [4,18,19].

In this study, based on the morphological characterization, molecular identification and pathogenicity assays, the pathogens associated with red rot symptoms were identified as *F. madaense* and *C. falcatum*. Of the 57 isolates obtained, 44 isolates (19 *F. madaense* and 25 *C. falcatum*) were from Yunnan Province, and 13 isolates (two *F. madaense* and 11 *C. falcatum*) were from Guangxi Province (Figure 4A). This may be attributed to the diverse cultivation of sugarcane varieties in Yunnan, where six varieties were sampled, compared with four varieties in Guangxi. Among the ten sampled varieties, *F. madaense* and *C. falcatum* were frequently isolated from Yuetang 93-159, and both fungi were also isolated from Yunzhe 081609, whereas only one of the two species (*F. madaense* or *C. falcatum*) was isolated in the other eight varieties (Figure 4B). Notably, *F. madaense* and *C. falcatum* were not isolated simultaneously from the same sample, and co-isolation was not observed among the samples analyzed in this study. However, it remains to be determined whether expanded sampling across sugarcane varieties and growing regions in China would identify other pathogens or co-infection events.

In recent years, *C. falcatum* and *Fusarium* species associated with sugarcane red rot symptoms have been reported. Dela Cueva et al. [20] reported that *F. sacchari* and *F. proliferatum* induce red rot in both the leaf midribs and stalks of sugarcane in the Philippines. Costa et al. [21] showed that *F. sacchari*, *F. proliferatum* and *F. madaense* cause cane stalk red rot in Brazil. Nevertheless, these *Fusarium* species are also pathogens of sugarcane pokkah boeng [37,40]. *F. madaense* was first isolated from groundnut (*Arachis hypogaea*) and sorghum (*Sorghum bicolor*) in Nigeria by Ezekiel et al. [36]. In recent years, *F. madaense* has been reported as a pathogen of multiple plants in Brazil, including *Brachiaria* spp., *Zea mays*, *Eleusine coracana*, *Pennisetum glaucum*, *Oryza sativa*, *S. bicolor*, and *S. officenarum* (pokkah boeng) [37]. Subsequently, Gunasinghe et al. [41] reported for the first time that *F. madaense* causes root and stalk rot on *S. bicolor* in Australia. To our knowledge, this is the first report of *F. madaense* associated with and inducing red rot symptoms in sugarcane leaf midribs and stalks in China.

Morphological and colony observations revealed no significant differences among *F. madaense* isolates. Phylogenetic analysis based on *EF-1α* and *RPB2* sequences further confirmed that no sequence polymorphisms were detected within these two DNA loci among the *F. madaense* isolates obtained in this study. *F. madaense* isolates from China clustered in the same clade with the *F. madaense* strain (CML 3586) isolated from sugarcane in Brazil and were phylogenetically closely related to *F. andiyazi*, which is consistent with previous reports by Ezekiel et al. [36] and Costa et al. [21]. In addition to *F. madaense*, *C. falcatum* showed distinct characteristics in morphological and genetic traits. This is the first study to classify *C. falcatum* isolates using morphological characteristics and multigene phylogenetic analysis in China. Thirty-six *C. falcatum* isolates were obtained from different sugarcane varieties and growing regions in China. This study revealed that *C. falcatum* is divided into a light type and dark type based on morphological characters in China, which is consistent with the previous reports from India, Thailand, Brazil, Bangladesh and Pakistan [5,9,10,21,42]. We also found that light-type colonies were whitish grey or greyish white, mostly raised and fluffy, with moderate to high sporulation; the dark type forms greyish green or dark grey colonies that are flat, less fluffy and have poor sporulation. Morphological and colonial differences indicated that there were significant differences among the *C. falcatum* isolates. Pathological assessment provided a preliminary finding that the light-type isolates are more virulent than the dark-type isolates; however, this finding requires validation with a larger number of isolates in future studies.

The ITS, *ACT* and *TUB2* genes have been widely used for the identification and phylogenetic analysis of the genus *Colletotrichum* [10,11,43]. Hossain et al. [5] reported that 41 *C. falcatum* isolates from Bangladesh clustered into a single clade, with no distinct geographical structuring evident within this clade based on multigene phylogenetic analysis. In this study, multigene phylogenetic analysis of 36 *C. falcatum* isolates from sugarcane in China was performed based on the ITS, *ACT* and *TUB2* sequences. The results show that isolates from Yunnan Province clustered into Clade I, whereas those from Guangxi Province clustered as Clade II. Guangxi and Yunnan Provinces are the first and second largest sugarcane-producing regions in China, both geographically located in southern China and sharing a contiguous border. However, the major cultivated sugarcane varieties differ between the two regions: Guangxi mainly grows varieties such as Guitang 42 and Guitang 44, while Yunnan primarily cultivates Yuetang 93-159, Yunzhe 081609, and ROC 22. Notably, no correlation was found between the genetic clades (Clade I and Clade II) and the morphological morphotypes (light type and dark type) in this study. A distinct geographical clustering was observed in Southwest China, which may be influenced by the diverse ecological conditions and cultivar compositions in the study area. In contrast, the hypothesis that the observed geographical differentiation of *C. falcatum* is partly related to the divergence in main cultivated varieties between Guangxi and Yunnan lacks supporting evidence and remains to be tested with additional data (e.g., seed cane movement records or population genetic analyses). Additionally, the molecular mechanisms underlying the virulence differences between light-type and dark-type *C. falcatum* remain unclear, which should be addressed in future studies through transcriptomic or genomic analyses.

In this study we confirmed that *C. falcatum* and the novel pathogen *F. madaense* induce leaf midribs and stalk red rot in Southwest China, and the *C. falcatum* isolates Cf16 and Cf1 showed higher stalk virulence than the tested *F. madaense* isolates. However, the pathogenicity test was performed exclusively on the susceptible variety Yuetang 93-159. Consequently, the observed virulence levels are specific to this variety and may differ from other sugarcane varieties with different genetic backgrounds. The results of this study can provide a scientific basis for resistance breeding, as well as the monitoring and management of this disease. The present study provides first-hand information on characterization and phylogenetic analyses of *F. madaense* and *C. falcatum* associated with sugarcane red rot disease in China.

## 5. Conclusions

Preliminary findings suggest that sugarcane red rot in Southwest China is caused by two fungal pathogens in the samples examined in this study: *C*. *falcatum* and the novel pathogen *F. madaense*. *C. falcatum* is the most frequently isolated species associated with sugarcane red rot, accounting for 63.2% of the total isolates in this study. Isolates of *C. falcatum* from sugarcane in Southwest China are classified into light and dark morphotypes, and preliminary findings from the tested isolates suggest that light-type isolates show significantly higher virulence than dark-type ones; however, further investigation with a larger number of isolates is required to confirm this observation. Additionally, the *C. falcatum* isolates Cf16 and Cf1 showed higher stalk virulence than the tested *F. madaense* isolates. Our findings thus lay a scientific foundation for the targeted monitoring, resistance breeding, and integrated management of sugarcane red rot in China. To our knowledge, this is the first report of *F*. *madaense* causing typical red rot symptoms on sugarcane in China.

## Figures and Tables

**Table 1:** *C. falcatum* and *F*. *madaense* isolates collected from different sugarcane varieties with symptoms of red rot in China.

Isolate	Species	Colony Colour ^1^	Varieties	Site of Collection	Region	Latitude (°N), Longitude (°E)	GenBank Accession No. ^2^				
							ITS	*ACT*	*TUB*	*EF-1α*	*RPB2*
**Cf1**	*C. falcatum*	Whitish grey	Yuetang 93-159	Menghai County	Yunnan	100°20′5″ E, 22°10′35″ N	OR523414	OR542860	OR542896	─	─
Cf2	*C. falcatum*	Greyish white	Yuetang 93-159	Menghai County	Yunnan	100°20′5″ E, 22°10′35″ N	OR523415	OR542861	OR542897	─	─
Cf3	*C. falcatum*	Whitish grey	Yuetang 93-159	Menghai County	Yunnan	100°20′5″ E, 22°10′35″ N	OR523416	OR542862	OR542898	─	─
Cf4	*C. falcatum*	Dark grey	Yuetang 93-159	Puer City	Yunnan	99°35′24″ E, 22°9′57″ N	OR523417	OR542863	OR542899	─	─
Cf5	*C. falcatum*	Greyish white	Yuetang 93-159	Puer City	Yunnan	99°35′24″ E, 22°9′57″ N	OR523418	OR542864	OR542900	─	─
Cf6	*C. falcatum*	Dark grey	Yuetang 93-159	Puer City	Yunnan	99°35′24″ E, 22°9′57″ N	OR523419	OR542865	OR542901	─	─
Cf7	*C. falcatum*	Greyish white	Yuetang 93-159	Puer City	Yunnan	99°35′24″ E, 22°9′57″ N	OR523420	OR542866	OR542902	─	─
Cf8	*C. falcatum*	Greyish white	Yuetang 93-159	Puer City	Yunnan	99°35′24″ E, 22°9′57″ N	OR523421	OR542867	OR542903	─	─
Cf9	*C. falcatum*	Whitish grey	Yuetang 93-159	Puer City	Yunnan	99°35′24″ E, 22°9′56″ N	OR523427	OR542873	OR542909	─	─
Cf10	*C. falcatum*	Greyish white	Yuetang 93-159	Puer City	Yunnan	99°35′24″ E, 22°9′56″ N	OR523438	OR542884	OR542920	─	─
Cf11	*C. falcatum*	Greyish white	Yuetang 93-159	Puer City	Yunnan	99°35′24″ E, 22°9′56″ N	OR523435	OR542881	OR542917	─	─
Cf12	*C. falcatum*	Greyish white	Yuetang 93-159	Puer City	Yunnan	99°35′24″ E, 22°9′56″ N	OR523436	OR542882	OR542918	─	─
Cf13	*C. falcatum*	Greyish white	Yuetang 93-159	Puer City	Yunnan	99°35′24″ E, 22°9′56″ N	OR523428	OR542874	OR542910	─	─
Cf14	*C. falcatum*	Dark grey	Yuetang 93-159	Lincang City	Yunnan	99°26′54″ E, 23°20′38″ N	OR523449	OR542895	OR542931	─	─
Cf15	*C. falcatum*	Greyish white	Yunzhe 081609	Puer City	Yunnan	99°49′9″ E, 23°2′12″ N	OR523430	OR542876	OR542912	─	─
**Cf16**	*C. falcatum*	Greyish white	Funong 10-14405	Mile City	Yunnan	103°19′55″ E, 23°55′27″ N	OR523440	OR542886	OR542922	─	─
Cf17	*C. falcatum*	Greyish green	Funong 10-14405	Mile City	Yunnan	103°19′55″ E, 23°55′27″ N	OR523441	OR542887	OR542923	─	─
Cf18	*C. falcatum*	Whitish grey	Funong 10-14405	Mile City	Yunnan	103°19′55″ E, 23°55′27″ N	OR523442	OR542888	OR542924	─	─
Cf19	*C. falcatum*	Greyish green	Funong 10-14405	Mile City	Yunnan	103°19′55″ E, 23°55′27″ N	OR523443	OR542889	OR542925	─	─
Cf20	*C. falcatum*	Greyish green	Funong 10-14405	Mile City	Yunnan	103°19′55″ E, 23°55′27″ N	OR523445	OR542891	OR542927	─	─
Cf21	*C. falcatum*	Greyish green	Funong 10-14405	Mile City	Yunnan	103°19′55″ E, 23°55′27″ N	OR523444	OR542890	OR542926	─	─
Cf22	*C. falcatum*	Greyish white	Chuantang 79-15	Lincang City	Yunnan	99°8′16″ E, 23°37′6″ N	OR523446	OR542892	OR542928	─	─
Cf23	*C. falcatum*	Greyish white	Chuantang 79-15	Lincang City	Yunnan	99°8′16″ E, 23°37′6″ N	OR523447	OR542893	OR542929	─	─
Cf24	*C. falcatum*	Greyish white	Chuantang 79-15	Lincang City	Yunnan	99°8′16″ E, 23°37′6″ N	OR523448	OR542894	OR542930	─	─
Cf25	*C. falcatum*	Greyish white	Guitang 44	Hechi City	Guangxi	108°32′19″ E, 24°24′41″ N	OR523422	OR542868	OR542904	─	─
Cf26	*C. falcatum*	Greyish white	Guitang 44	Hechi City	Guangxi	108°7′11″ E, 24°46′44″ N	OR523431	OR542877	OR542913	─	─
Cf27	*C. falcatum*	Greyish white	Guitang 44	Hechi City	Guangxi	108°7′11″ E, 24°46′44″ N	OR523437	OR542883	OR542919	─	─
Cf28	*C. falcatum*	Greyish white	Guitang 44	Wuzhou City	Guangxi	110°39′55″ E, 24°7′58″ N	OR523426	OR542872	OR542908	─	─
Cf29	*C. falcatum*	Greyish white	Guitang 44	Wuzhou City	Guangxi	110°39′55″ E, 24°7′58″ N	OR523425	OR542871	OR542907	─	─
Cf30	*C. falcatum*	Greyish white	Liucheng 03-1137	Hechi City	Guangxi	108°7′20″ E, 24°46′35″ N	OR523423	OR542869	OR542905	─	─
Cf31	*C. falcatum*	Greyish white	Liucheng 03-1137	Hechi City	Guangxi	108°7′20″ E, 24°46′35″ N	OR523439	OR542885	OR542921	─	─
Cf32	*C. falcatum*	Greyish green	Liucheng 03-1137	Hechi City	Guangxi	108°7′20″ E, 24°46′35″ N	OR523434	OR542880	OR542916	─	─
**Cf33**	*C. falcatum*	Greyish green	Liucheng 03-1137	Wuzhou City	Guangxi	110°39′41″ E, 24°8′5″ N	OR523424	OR542870	OR542906	─	─
Cf34	*C. falcatum*	Greyish green	Guitang 44	Hechi City	Guangxi	108°7′11″ E, 24°46′44″ N	OR523432	OR542878	OR542914	─	─
Cf35	*C. falcatum*	Greyish white	Guitang 44	Hechi City	Guangxi	108°7′11″ E, 24°46′44″ N	OR523433	OR542879	OR542915	─	─
Cf36	*C. falcatum*	Greyish green	Yuetang 93-159	Puer City	Yunnan	99°35′24″ E, 22°9′56″ N	OR523429	OR542875	OR542911	─	─
FM1	*F. madaense*	Pale salmon	Yunzhe 081609	Kaiyuan City	Yunnan	103°15′52″ E, 23°42′22″ N	─	─	─	PP796366	PP691538
**FM2**	*F. madaense*	White	Yuetang 93-159	Puer City	Yunnan	99°41′27″ E, 22°26′7″ N	─	─	─	PP796367	PP691539
FM3	*F. madaense*	White	ROC 22	Lincang City	Yunnan	99°21′19″ E, 23°26′56″ N	─	─	─	PP796368	PP691540
FM4	*F. madaense*	White	Yunzhe 081609	Lincang City	Yunnan	100°2′9″ E, 24°4′21″ N	─	─	─	PP796369	PP691541
FM5	*F. madaense*	Pale salmon	Liucheng 05-136	Hechi City	Guangxi	108°6′53″ E, 24°36′31″ N	─	─	─	PP796370	PP691542
**FM6**	*F. madaense*	Pale salmon	Guitang 42	Hechi City	Guangxi	108°17′19″ E, 24°33′15″ N	─	─	─	PP796371	PP691543
FM7	*F. madaense*	White	Yuetang 93-159	Puer City	Yunnan	99°35′15″ E, 22°10′33″ N	─	─	─	PP796372	PP691544
FM8	*F. madaense*	White	Yuetang 93-159	Puer City	Yunnan	99°41′27″ E, 22°26′7″ N	─	─	─	PP796373	PP691545
**FM9**	*F. madaense*	White	ROC 22	Lincang City	Yunnan	100°1′31″ E, 24°4′37″ N	─	─	─	PP796374	PP691546
FM10	*F. madaense*	White	Yuetang 93-159	Puer City	Yunnan	99°50′35″ E, 23°5′31″ N	─	─	─	PP796375	PP691547
FM11	*F. madaense*	White	Yuetang 93-159	Puer City	Yunnan	99°50′35″ E, 23°5′31″ N	─	─	─	PP796376	PP691548
FM12	*F. madaense*	White	Yuetang 93-159	Puer City	Yunnan	99°50′35″ E, 23°5′31″ N	─	─	─	PP796377	PP691549
FM13	*F. madaense*	White	Yingyu 91-59	Lincang City	Yunnan	99°27′3″ E, 23°21′43″ N	─	─	─	PP796378	PP691550
FM14	*F. madaense*	White	Yunzhe 081609	Lincang City	Yunnan	100°1′23″ E, 24°4′39″ N	─	─	─	PP796379	PP691551
FM15	*F. madaense*	White	Yunzhe 081609	Lincang City	Yunnan	100°1′25″ E, 24°4′36″ N	─	─	─	PP796380	PP691552
FM16	*F. madaense*	Pale salmon	Yunzhe 081609	Kaiyuan City	Yunnan	103°15′52″ E, 23°42′22″ N	─	─	─	PP796381	PP691553
FM17	*F. madaense*	Pale salmon	Yunzhe 081609	Kaiyuan City	Yunnan	103°15′52″ E, 23°42′22″ N	─	─	─	PP796382	PP691554
FM18	*F. madaense*	White	Yunzhe 081609	Lincang City	Yunnan	100°1′31″ E, 24°4′49″ N	─	─	─	PP796383	PP691555
FM19	*F. madaense*	White	ROC 22	Lincang City	Yunnan	99°24′18″ E, 23°27′6″ N	─	─	─	PP796384	PP691556
FM20	*F. madaense*	Pale salmon	Yuetang 93-159	Kaiyuan City	Yunnan	103°15′51″ E, 23°42′21″ N	─	─	─	PP796385	PP691557
FM21	*F. madaense*	Pale salmon	Yuetang 93-159	Kaiyuan City	Yunnan	103°15′51″ E, 23°42′21″ N	─	─	─	PP796386	PP691558

^1^ Colony color: The colony characteristics of this fungus on PDA; light type: greyish white or whitish grey; dark type: greyish green or dark grey. ^2^ ITS: internal transcribed spacer; *ACT*: *Actin*; *TUB2*: *β-tubulin*; *EF-1α*: the translation elongation factor-1α gene; *RPB2*: the second largest subunit of RNA polymerase II. The representative isolates used for the pathogenicity test and micromorphological characteristics are highlighted in bold.

**Table 2:** Information of reference strains used for the phylogenetic analyses in this study.

Species	Strains	Host	Country	GenBank Accession No. ^1^				
				ITS	*ACT*	*TUB*	*EF-1α*	*RPB2*
*C. falcatum*	I-1	*Saccharum officinarum*	Bangladesh	MN636336	MN643114	MN643093	─	─
*C. falcatum*	I-2	*S. officinarum*	Bangladesh	MK850182	MK867379	MK867399	─	─
*C. falcatum*	I-3	*S. officinarum*	Bangladesh	MN636355	MN643115	MN643094	─	─
*C. falcatum*	I-5	*S. officinarum*	Bangladesh	MK850183	MK867380	MK867400	─	─
*C. falcatum*	CML 4078	*S. officinarum*	Brazil	MW471110	MW455491	Unknown	─	─
*C. falcatum*	CML 4080	*S. officinarum*	Brazil	MW471111	MW455492	Unknown	─	─
*C. falcatum*	CML 4081	*S. officinarum*	Brazil	MW471112	MW455493	Unknown	─	─
*C. falcatum*	CML 3861	*S. officinarum*	Brazil	MW471108	MW455489	Unknown	─	─
*C. falcatum*	CML 4075	*S. officinarum*	Brazil	MW471109	MW455490	Unknown	─	─
*C. falcatum*	COUFAL0263	*S. officinarum*	Brazil	MT796068	Unknown	MT778880	─	─
*C. falcatum*	COUFAL0264	*S. officinarum*	Brazil	MT796069	Unknown	MT778881	─	─
*C. falcatum*	COUFAL0265	*S. officinarum*	Brazil	MT796070	Unknown	MT778882	─	─
*C. falcatum*	COUFAL0266	*S. officinarum*	Brazil	MT796071	Unknown	MT778883	─	─
*C. falcatum*	Cf86032C	*S. officinarum*	India	FJ002036	FJ008081	Unknown	─	─
*C. falcatum*	cf01	*S. officinarum*	India	KU220959	Unknown	Unknown	─	─
*C. falcatum*	Cf-06	*S. officinarum*	India	AB242414	Unknown	Unknown	─	─
*C. falcatum*	RR01	*S. officinarum*	India	KU220961	Unknown	Unknown	─	─
*C. falcatum*	RR03	*S. officinarum*	India	KU220963	Unknown	Unknown	─	─
*C. falcatum*	cfCHA	*S. officinarum*	India	KP869833	Unknown	Unknown	─	─
*C. falcatum*	cfKAM	*S. officinarum*	India	KP869832	Unknown	Unknown	─	─
*C. falcatum*	CoC671	*S. officinarum*	India	KP184444	Unknown	Unknown	─	─
*C. falcatum*	SUCF04	*S. officinarum*	Pakistan	MT197390	Unknown	Unknown	─	─
*C. falcatum*	Unknown	*S. officinarum*	India	AY944749	Unknown	Unknown	─	─
*C. falcatum*	Unknown	*S. officinarum*	India	AY944745	Unknown	Unknown	─	─
*C. falcatum*	Unknown	*S. officinarum*	India	AY944744	Unknown	Unknown	─	─
*C. endophytum*	CGMCC 3.15108	*Bletilla ochracea*	China	JX625177	KC843533	JX625206	─	─
*C. bletillum*	CGMCC 3.15117	*B. ochracea*	China	JX625178	KC843542	JX625207	─	─
*C. tofieldiae*	CGMCC 3.15118	*B. ochracea*	China	JX625176	KC843541	JX625205	─	─
*C. gloeosporioides*	IMI 356878	*Citrus sinensis*	Italy	JX010152	JX009531	JX010445	─	─
*C. fructicola*	C1253.2	*Limonium sinuatum*	Israel	JX010167	JX010388	JX009491	─	─
*Monilochaetes infuscans*	CBS 869.96	Unknown	Unknown	JQ005780	JQ005864	JQ005843	─	─
*F. andiyazi*	CBS 119856	*Sorghum grain*	Ethiopia	─	─	─	MN533989	MN534286
*F. andiyazi*	CBS 119857	*Sorghum bicolor* soil debris	South Africa	─	─	─	MN193854	LT996138
*F. bilaiae*	MFG 60364	*Helianthus annuus*	Russian Federation	─	─	─	MW286112	MW286116
*F. brevicatenulatum*	CBS 404.97	*Striga asiatica*	Madagascar	─	─	─	MN533995	MN534295
*F. concentricum*	CBS 450.97	*Musa fruit*	Costa Rica	─	─	─	AF160282	JF741086
*F. fujikuroi*	CBS 221.76	*Oryza sativa* culm	China, Taiwan	─	─	─	MN534010	KU604255
*F. fujikuroi*	CBS 257.52	*O. sativa*	Japan	─	─	─	MW402119	MW402812
*F. globosum*	NRRL 26131	*Zea mays*	South Africa	─	─	─	KF466417	KF466406
*F. inflexum*	NRRL 20433	*Vicia faba*	USA	─	─	─	AF008479	JX171583
*F. madaense*	CBS 146648	*Arachis hypogaea*	Nigeria	─	─	─	MW402095	MW402761
*F. madaense*	CBS 146651	*S. bicolor*	Nigeria	─	─	─	MW402096	MW402762
*F. madaense*	CBS 146656	*A. hypogaea*	Nigeria	─	─	─	MW402097	MW402763
*F. madaense*	CBS 146669	*A. hypogaea*	Nigeria	─	─	─	MW402098	MW402764
*F. madaense*	CML 2791	*S. bicolor*	Brazil	─	─	─	MK895716	Unknown
*F. madaense*	CML 3044	*Brachiaria brizantha*	Brazil	─	─	─	MK895713	Unknown
*F. madaense*	CML 3586	*S. officinarum*	Brazil	─	─	─	MH187929	MH187912
*F. madaense*	CML 3656	*Urochloa brizantha*	Brazil	─	─	─	MT901357	Unknown
*F. madaense*	CML 3875	*S. bicolor*	Brazil	─	─	─	MK895722	Unknown
*F. madaense*	CML 4117	*S. officinarum*	Brazil	─	─	─	MW455466	Unknown
*F. madaense*	CML 4118	*S. officinarum*	Brazil	─	─	─	MW455467	Unknown
*F. madaense*	CML 4121	*S. officinarum*	Brazil	─	─	─	MW455468	Unknown
*F. madaense*	CML 4194	*S. officinarum*	Brazil	─	─	─	MW455473	Unknown
*F. mangiferae*	CBS 120994	*Mangifera indica*	Israel	─	─	─	MN534017	MN534271
*F. musae*	CBS 624.87	*Musa sapientum*	Honduras	─	─	─	FN552086	MW402772
*F. napiforme*	CBS 748.97	*Pennisetum typhoides*	Namibia	─	─	─	MN193863	MN534291
*F. napiforme*	CBS 135141	Clinical	Unknown	─	─	─	MW402045	MW402797
*F. oxysporum*	NRRL 22902	*Pseudotsuga menziesii*	USA	─	─	─	AF160312	LT575065
*F. proliferatum*	CBS 480.96	Soil	Papua New	─	─	─	MN534059	MN534272
*F. pseudoanthophilum*	CBS 745.97	*Z. mays*	Zimbabwe	─	─	─	MW402148	MW402820
*F. ramigenum*	NRRL 25208	*Ficus carica*	USA	─	─	─	KF466423	KF466412
*F. ramigenum*	CBS 526.97	*F. carica*	USA	─	─	─	MN534032	MN534292
*F. siculi*	CPC 27188	*C. sinensis*	Italy	─	─	─	LT746214	LT746327
*F. sublunatum*	NRRL 13384	Soil	Costa Rica	─	─	─	OM160871	OM160850
*F. verticillioides*	CBS 218.76	*Z. mays*	Germany	─	─	─	MW402113	MW928835
*F. xylarioides*	CBS 749.79	*Coffea canephora*	Guinea	─	─	─	MN534049	MN534259

^1^ ITS: internal transcribed spacer; *ACT*: *Actin*; *TUB2*: *β-tubulin*; *EF-1α*: the translation elongation factor-1α gene; *RPB2*: the second largest subunit of RNA polymerase II.

**Table 3:** Micromorphology of the conidia and pathogenicity of the representative *C. falcatum* and *F. madaense* isolates from China.

Inoculum	Colony Color (on PDA)	Sporulation ^1^	Conidial Size (µm) ^2^				Disease Reactions	
			Conidia/Microconidia		Macroconidia		Infected Leaf Midrib (mm) ^3^	Infected Stalk ^4^
			Length	Width	Length	Width		
Cf1	Whitish grey	++	32.07 ± 2.49 a	5.56 ± 0.23 c	—	—	64.33 ± 11.42 a	2–3 nodes, 2.56 ± 0.53 a
Cf16	Greyish white	+++	29.93 ± 1.63 b	5.93 ± 0.39 b	—	—	70.33 ± 7.66 a	3–4 nodes, 3.44 ± 0.53 a
Cf33	Greyish green	+	29.54 ± 1.43 b	6.56 ± 0.26 a	—	—	27.11 ± 1.97 c	1–2 node, 1.56 ± 0.53 ab
FM2	White	+++	15.13 ± 5.17 a	3.00 ± 0.68 a	36.36 ± 7.23 a	3.66 ± 0.31 a	39.89 ± 3.98 bc	0–1 node, 0.67 ± 0.50 b
FM6	Pale salmon	+++	13.88 ± 5.48 a	2.88 ± 0.65 a	35.77 ± 9.27 a	3.69 ± 0.38 a	50.33 ± 4.09 ab	0–1 node, 0.89 ± 0.33 b
FM9	White	+++	14.56 ± 6.35 a	2.85 ± 0.68 a	35.25 ± 6.84 a	3.60 ± 0.26 a	43.44 ± 5.70 bc	0–1 node, 0.78 ± 0.44 b

^1^ Sporulation: ‘+’ poor; ‘++’ medium; ‘+++’ high. ^2,3^ Mean ± standard deviation. Values within the same column followed by the same letters mean that they are not significantly different based on the Kruskal–Wallis test with Dunn’s post hoc test at *p* < 0.05. For conidial size, comparisons were made only within *C. falcatum* isolates or within *F. madaense* isolates; no cross-genus comparisons were performed. ^3^ Average lesion length. ^4^ Lesion transgression of the inoculated node: 0 = no node transgression; 1 = transgression of one node; 2 = transgression of two nodes; 3 = transgression of three nodes; 4 = transgression of four nodes.

## Data Availability

The original contributions presented in this study are included in the article. Further inquiries can be directed to the corresponding authors. The DNA sequence data generated for this study have been deposited in the GenBank database under the accession numbers listed in Table 1.
